# Considerations in the Design of Non-Clinical Development Programmes to Support Non-Viral Genetically Modified Mesenchymal Stromal Cell Therapies

**DOI:** 10.3390/pharmaceutics13060823

**Published:** 2021-06-02

**Authors:** Valeria Iansante, Andrew Brooks, Lee Coney

**Affiliations:** Cell and Gene Therapy Catapult, 12th Floor Tower Wing, Guy’s Hospital, Great Maze Pond, London SE1 9RT, UK; iansante.valeria@gmail.com (V.I.); andrew.brooks@ct.catapult.org.uk (A.B.)

**Keywords:** mesenchymal stromal cells, mesenchymal stem cells, MSC, genetically modified, non-viral approaches, gene therapy, cell therapy, non-clinical strategy, preclinical studies, regulatory requirements

## Abstract

Due to their immune suppressive pharmacology, regenerative capacity, and immune privileged status, mesenchymal stromal cells (MSCs) are an attractive cell type to treat a variety of diseases. Genetically engineered MSCs are currently in non-clinical and clinical development for a wide range of applications including the delivery of pro-drugs and therapeutic proteins or modified to enhance their regenerative potential. Unmodified MSCs have been shown to have good safety profiles in clinical development. The introduction of exogenous transgenes introduces possible additional risks that need to be assessed in non-clinical studies prior to initiating clinical studies. The use of ex vivo non-viral genetic modification approaches potentially reduces the risks associated with viral vector transfection approaches, including the potential for cell transformation. This review provides an overview of the regulatory-compliant non-clinical proof-of-concept and safety studies required to take MSC-based gene therapy products from the bench to the clinic.

## 1. Introduction

Mesenchymal stromal cells (MSCs) are a heterogenous population of cells that can be isolated from various supportive stromal tissues and expanded in culture. They can be obtained from several sources with bone marrow, adipose tissue, and umbilical cord currently being the most common. Despite the term mesenchymal *stem* or *stromal* cells being used interchangeably, a recent position statement by the International Society for Cell & Gene Therapy (ISCT^®^) clarified that MSCs are stromal cells with specific secretory [1], immunomodulatory [2] and homing properties [3], and are defined by minimal criteria which include: (i) being plastic adherent; (ii) expressing specific markers (CD73, CD90, and CD105); (iii) lacking expression of haematopoietic and endothelial markers (CD11b, CD14, CD19, CD34, CD45, CD79a, and HLA-DR); and (iv) being capable of differentiation under defined in vitro conditions into cells of the mesenchymal lineages, i.e., adipocyte, chondrocyte, and osteoblast lineages [4]. Mesenchymal *stem* cells are a rarer stem cell subpopulation with a demonstrable progenitor cell functionality of self-renewal and differentiation [5]. Due to the lack of exclusive markers for stemness, rigorous functional in vitro and in vivo evidence should be used to demonstrate the progenitor self-renewal and differentiation properties of mesenchymal stem cells e.g., clonogeneic studies [6].

MSCs have been widely investigated for their use as cell therapies in several inflammatory and degenerative diseases, due to their immunomodulatory, anti-inflammatory, and pro-regenerative properties. Several studies have shown that MSCs can modulate both adaptive [7] and innate [2] immune responses, and show preferential homing to sites of inflammation in damaged tissues [8,9,10]. In pro-inflammatory environments, MSCs produce anti-inflammatory mediators, including indoleamine 2,3-dioxygenase (IDO), TNF-stimulated gene 6 (TSG-6), and the transforming growth factor-β (TGF-β), which induce anti-inflammatory M2 macrophages and regulatory T cells (Tregs), inhibit the extravasation of leukocytes, and negatively regulate the activation and proliferation of T cells [2,11,12,13]. In addition to their immunomodulatory role, MSCs have also been shown to induce pro-regenerative effects, mainly related to their paracrine ability and their secretion of microvesicles, exosomes, and growth factors, including hepatocyte growth factor (HGF), fibroblast growth factor (FGF), and vascular endothelial growth factor (VEGF) [14,15]. Considering their immunomodulatory and pro-regenerative effects, investigations regarding the role of MSCs in tumour development have also been carried out. However, the ability of MSCs to promote or inhibit the activities of transformed cells remains controversial. Evidence shows that MSCs migrate towards tumour sites, where they seem to have the ability to exert both stimulatory and inhibitory effects on cancer cell growth and invasion potential, depending upon the tumour type, stage, and possibly the composition of the MSC population itself [16].

An additional property of MSCs that makes them attractive for cell therapy is their immune evasive ability, as they express low levels of MHC class I and are negative for MHC class II. Together this allows for their use in allogeneic transplantation. The expression of MHC class I and II in MSCs can be induced by some stimuli (e.g., IFN-γ) or upon differentiation into mature cell types [17]. The original perception of MSC as immune privileged has more recently been revisited considering that mismatched MSCs show some degree of immunogenicity, although at a much lower level than the level observed with other cell types [18].

MSCs have largely been investigated in clinical trials for the treatment of diseases such as graft-versus-host disease [19], autoimmunity [20], ischemic heart disease [21], inflammatory conditions [22], and infectious diseases, including coronavirus-induced disease (COVID-19) [23]. Encouraging results and good safety profiles have led to a growing number of studies. Alofisel, an allogeneic adipose-derived MSC product, received marketing authorisation approval in Europe in 2018 for the treatment of complex perianal fistulas in adult patients with Crohn’s disease (an inflammatory condition of the gut) [24].

Due to their availability, relatively simple isolation, ex vivo expansion, low immunogenicity, and overall safety observed in the clinic, further clinical applications of MSCs are currently being explored. Genetic modifications have been proposed as a tool to enhance the regenerative properties of MSCs, or to use them as carriers for the delivery of pro-drugs, therapeutic proteins, and enzymes. While viral gene delivery is well established and highly efficient, it still faces potential safety risks, mainly due to possible insertional mutagenesis events that can result in cell transformation. Recently, it was reported that a patient treated in 2016 on a compassionate use basis with Strimvelis^®^, a gammaretroviral engineered haematopoietic stem cell (HSC) product approved by the European Medicines Agency (EMA) for the treatment of ADA-SCID, an adenosine deaminase (ADA) deficiency caused by mutations in the *ADA* gene leading to severe combined immunodeficiency (SCID), has been diagnosed with lymphoid T-cell leukaemia, possibly caused by an insertional event related to the treatment [25]. Although MSCs have not shown any signs of tumourigenicity thus far, transduction with recombinant integrating viral vectors could affect their safety profiles. Alternative methods to modify MSCs ex vivo using non-viral approaches, such as microinjection, electroporation, and nanocarriers [26], are being investigated clinically for a wide range of diseases, including ischemic stroke [27], traumatic brain injury [28], cancer [29], and COVID-19 [30].

The aim of this manuscript is to provide an overview of the non-clinical studies required to enable the translation of genetically modified MSCs with non-viral approaches to first-in-human (FiH) clinical studies in line with the current regulatory guidelines. Whilst we recognise that components of the Chemistry-Manufacturing-Control (CMC) activities also contribute to the understanding of both the safety profiles and quality attributes of these and other advanced therapy medicinal products (ATMPs), we intend to limit the scope of this manuscript to a description of the main considerations related to the non-clinical aspects of MSC-based non-viral gene therapy product development.

## 2. Clinical Landscape of MSCs Genetically Modified with Non-Viral Methods

Due to their inherent regenerative and multipotent nature, MSCs are being extensively investigated in clinical trials. Whilst the majority of MSC-based clinical trials utilise unmodified MSCs as their therapeutic product, a small number use genetically modified MSCs that overexpress a desired recombinant protein. Genes of interest can be delivered into MSCs either in vivo or ex vivo, using viral or non-viral methods. Currently, the most common method of gene delivery is performed ex vivo using lentiviral vector systems, however, non-viral based approaches are also being investigated. One of the most advanced non-viral genetically modified products is SB623 (SanBio), which recently completed Phase II trials in ischemic stroke patients with fixed motor deficits.

Of the 21 genetically modified MSC trials identified in a GlobalData extract performed on the 15 March 2021, oncology (10) was the largest therapeutic area accounting for approximately half of the genetically modified MSC clinical trials. Approximately half of the trials were Phase I/II with the most advanced trials being Phase II. Non-viral genetically modified MSCs accounted for six clinical trials with an even spread of therapeutic areas being investigated: cardiovascular (2), CNS (1), oncology (1), respiratory (1), and infectious disease (1). Half of these trials were Phase I/II with the most advanced also being in Phase II development. Of the six trials, four non-viral genetically modified MSC products were identified, SB623, Descartes-30, MSC/HGF, and MSC-IFNβ, and are outlined in Table 1. Trials that did not have a public database ID (such as EudraCT or Clinical Trials) were removed from the extract.

### 2.1. SB623

SB623 is comprised of allogeneic adult bone-marrow-derived MSCs that are transiently transfected with a plasmid encoding the intracellular domain of human Notch-1. This allows ligand independent activation of the highly conserved Notch-1 signalling pathway which regulates many important cellular processes, including neuronal migration [31], vascularisation [32,33,34], immunosuppression [35,36], and inhibits apoptosis [37,38]. SB623 cells have been shown to promote a neurosupportive extracellular matrix [39] and the secretion of trophic [40] and chemotactic factors, which together aim to support damaged neural cells. The expression of the intracellular domain of Notch-1 also aims to lower the potential for these MSCs to differentiate into bone [41], cartilage [42], or adipose cells [43]. SanBio completed Phase II trials for SB623 to treat patients with ischemic stroke (Clinical trial: NCT02448641) in 2018 [44,45] and traumatic brain injury (Clinical trial: NCT02416492) in 2019 [28,43].

### 2.2. Descartes-30

Descartes-30 is an allogeneic MSC product transfected with Cartesian’s proprietary RNA Armory^SM^ platform. This ex vivo platform transiently transfects MSCs with mRNAs encoding two DNases that are secreted and act synergistically to specifically degrade Neutrophil Extracellular Traps (NETs), networks of extracellular fibres composed of DNA, histones, and granule-derived enzymes. Although initially described as microbicidal, they have since been implicated in a variety of diseases including acute respiratory distress syndrome (ARDS) and autoimmune diseases. A Phase I/II clinical trial (Clinical trial: NCT04524962) in patients with moderate-to-severe acute respiratory distress syndrome (ARDS) and COVID-19 is currently ongoing/recruiting [30].

### 2.3. MSC/HGF

Autologous bone marrow derived MSCs transfected ex vivo with cDNA encoding the human hepatocyte growth factor (HGF) were investigated in a Phase I/II clinical trial (Clinical trial: NCT01977131) [46] which was completed in 2013. In this study, MSC/HGF cells were infused intravenously weekly for three consecutive weeks at a dose of 2 × 10^6^ cells/kg into patients with silicosis [47], a fibrotic inflammatory lung disease caused by the inhalation of crystalline silica particles.

### 2.4. MSC-INFβ

A Phase I study (Clinical trial: NCT02530047) [29] investigated allogeneic MSCs electroporated ex vivo with a plasmid vector encoding the human interferon beta (IFNβ) gene. Type I interferons (IFN-α and IFN-β) are well known to have anti-tumour effects through the activation of the JAK/STAT signalling pathway and the subsequent transcription of various interferon-stimulated genes. Indeed, IFN-β signalling correlates with improved patient survival and repressed cancer stem cells in triple-negative breast cancer [48] and increasing tumour sensitivity to gemcitabine [49]. In this study, ovarian cancer patients received 4 weekly intraperitoneal infusions (IPI) of 1 × 10^5^ MSC-IFNβ [50].

## 3. Non-Clinical Development

The translation of advanced therapy medicinal products (ATMPs) to the clinic requires that some aspects of the therapeutics are investigated non-clinically including, at a minimum, efficacy, the clinical route of administration (ROA), dose, and safety. Due to the novelty and biological complexity of ATMPs, regulatory authorities suggest that a risk-based approach should be undertaken to identify the relevant clinical safety risks associated with each product, such as toxicity, unwanted biodistribution, tumourigenicity, and immunogenicity [51]. Once the risks are identified, specifically designed non-clinical studies are performed to assess the extent of these risks in suitable in vitro, in vivo and/or ex-vivo models that can predict clinical outcomes. In some cases, it is not possible to investigate all clinical risks in suitable non-clinical models, for example when a suitable animal model is not available. In other instances, the results of the non-clinical studies may predict a high probability that the identified risks will be replicated clinically, therefore a clinical mitigation plan and a strategy to support clinical translation should be established.

Each ATMP has a unique set of risks based on the biological characteristics of the product, therefore a case-by-case approach is always undertaken when designing an appropriate non-clinical strategy.

Ex vivo, non-viral modified MSCs are typically considered as gene therapy medicinal products (GTMPs). Although MSCs are relatively well characterised in the clinic, the addition of genetic modifications adds a level of complexity that should be considered when non-clinical studies are designed. Minimum non-clinical data requirements for FiH studies with ATMPs have been described in a draft guideline published by the European Medicines Agency in 2019 [52]. According to these regulatory requirements, an ideal non-clinical study programme for an ATMP should (i) demonstrate proof-of-concept in a relevant model; (ii) support the use of the clinical administration route and application procedure; (iii) support the selection of safe and biologically effective starting doses; and (iv) provide appropriate safety data to inform clinical monitoring.

### 3.1. Animal Selection

The selection of a relevant animal model for non-clinical efficacy and safety studies depends on several considerations. The animal model should be clinically relevant and predictive of the patient population in terms of disease status, anatomy, and target organ physiology. Furthermore, the selected species should allow the assessment of efficacy and/or safety. To do so, the medicinal product and transgene product(s) should be pharmacologically active in the selected species for several reasons. First, to observe pharmacology the product should be fully functional in the selected species, able to reach and recognise its target, and to induce the desired pharmacodynamic response. Secondly, unlike many novel chemical entities, the dose limiting effects of ATMPs may be due to exaggerated pharmacological effects. As such, the potential dose limiting effects can only be observed and characterised when the product is pharmacologically active in the selected animal model. Furthermore, possible differences in cell biodistribution and persistence in humans and the selected non-clinical model should be acknowledged and minimised when possible. If no feasible animal models are available, animal equivalent products and/or analogous species-specific transgene(s) can also be considered [53]. The principles of the 3R’s (reduction, replacement, refinement) should be incorporated into the planning of in vivo animal studies. Furthermore, these studies should be carefully planned to ensure the generation of robust and reproducible data. Where appropriate, animal testing should be replaced by in vitro or ex vivo studies.

The chosen animal model(s) may include wild-type, immunocompromised, knock-out, knock-in, humanised, or transgenic animals depending on the aim of the study [54]. Studies in only one pharmacologically relevant animal species are usually considered sufficient for most ATMPs if the model is considered predictive and the data are translatable to the clinic. However, the use of multiple animal species or strains may be necessary to address all of the appropriate safety aspects, and are generally determined on a case-by-case basis [52].

The use of the same animal model in both the toxicology investigations and the pharmacokinetic studies (i.e., biodistribution, migration, persistence, and clearance) may be beneficial, as it allows the correlation of the biodistribution of the ATMP with the observed toxicity signals. In some instances, the safety data can be collected from disease models to mimic the clinical use and to capture the safety concerns related to the product and the administration procedure. However, as for all ATMPs, this should be decided on a case-by-case basis. For example, the non-clinical studies supporting Alofisel were performed using different animal models for proof-of-concept and safety/biodistribution studies [55]. Proof-of-concept efficacy studies were performed in an experimental model of colitis, the trinitrobenzenesulfonic acid (TNBS)-induced colitis mouse model, which mimics the inflammatory environment seen in the gut in the presence of Crohn’s disease and human MSCs were transplanted. For biodistribution, toxicology, and tumourigenicity studies, the immunocompromised athymic nude rat model was used. This was conducted to minimise the risk of immune rejection and to maximise in vivo cell persistence, which results in an improved detection of the possible adverse effects [55].

### 3.2. Efficacy

Non-clinical efficacy studies have the primary objective to establish the feasibility and rationale for the use of an investigational ATMP in the targeted patient population. In addition, they should investigate the pharmacologically effective dose range (i.e., minimally effective dose and optimal biological dose), the most effective ROA and dosing regimen, and the characterisation of the putative mechanism of action. Whilst it is not necessary to use the final clinical product in these studies, one representative of the possible final clinical product should be used.

Pharmacodynamic studies are required to establish the potential clinical effect or related biological effect/molecular mechanism of action of the product. The expression and the production of the correct transgene product must be demonstrated as well as its capacity to function as intended. Evidence of functionality can be obtained using a combination of in vitro and in vivo strategies. Non-clinical studies aiming to demonstrate efficacy in a target disease, either in vitro or in vivo, do not need to be performed under Good Laboratory Practice (GLP). Where relevant it may be helpful to include toxicity and/or biodistribution endpoints in efficacy studies.

The proposed mechanism of action of Alofisel is based on the capacity of the expanded Adipose Stem Cells (eASC) to reduce inflammation, which is the main cause of fistula formation in Crohn’s disease. In vitro and in vivo studies were carried out to demonstrate the efficacy of the product. In vitro, studies focussed on the characterisation of anti-inflammatory cytokines released by these cells and their ability to induce Tregs. However, the role of these eASCs in modulating the inflammatory response in TNBS-induced colitic mice was investigated in vivo. For this, colitic mice received an intraperitoneal (i.p.) injection with either 3 × 10^5^ or 1 × 10^6^ eASC 12 h after an intrarectal injection of TNBS. A significant dose dependent improvement in the overall survival and body weight loss was observed with eASC treatment relative to control. Furthermore, a significant reduction in CD4+ CD11b+ infiltrating macrophages was also observed in the eASC treated mice. Importantly, compared to untreated colitic mice, a reduction in the concentration of inflammatory cytokines (TNF-α, IFN-γ, IL-6, IL-1β and IL-12) as well as the macrophage inhibitory protein 2 (MIP-2) was observed with the eASC treatment, further supporting the claim that eASCs have a significant anti-inflammatory role [55].

SB623, allogeneic MSCs expressing Notch-1 have been shown to be effective for the treatment of ischemic strokes in non-clinical in vitro and in vivo models. In vitro, a stroke model was established using primary rat cortical neurons or rat hippocampal brain slices induced to an ischemic state by oxygen and glucose deprivation (OGD). The cells were co-cultured with human MSCs or SB623 cells and signs of neural cell damage/death were measured, and neurotrophic factors released by MSCs and/or SB623 were assessed. A significant reduction in neural cell damage, measured by LDH release, was observed when neurons/slices were co-cultured with either MSCs or SB623 cells compared to untreated conditions (*p* < 0.05) [40]. Additionally, 11 neurotrophic factors secreted by MSCs and/or SB623 cells were identified (BMP-4, DKK-1, FGF-7, HB-EGF, IL-6, IL-8, MCP-1, MMP-1, PDGF-AA, VEGF, and HGF), presumably involved in the mechanism of action exerted by MSCs and SB623 cells in rescuing ischemic neurons [40]. In vivo, SB623 cells or their rat equivalent cells were transplanted intrastriatally into a rat model of chronic ischemic stroke, obtained by the transient occlusion of the middle cerebral artery (MCAo). Significant improvements in locomotor and neurological function were detected in stroke rats that received 1 × 10^5^ and 2 × 10^5^ rat cells, but not in those that received 0.4 × 10^5^ BMSCs. Similarly, when a high dose of human SB623 cells were transplanted (1.8 × 10^5^), significant improvements in both locomotor and neurological function were observed from day 7 to day 28 post-transplantation. Of note, all animals were immunosuppressed (10 mg/kg cyclosporin, i.p., daily) throughout the study. Human transplanted cells were still detected in the ischemic rat striatum at 4 weeks post-transplant, most probably due to the use of immunosuppression. Additionally, some safety endpoints were added to the study, consisting of a gross histological examination of the brain. No signs of donor-derived tumours or ectopic tissue formations were observed [56].

More recently, treatment with SB623 has also been proposed for critical limb threatening ischemia (CLTI) and tested non-clinically in Sprague-Dawley rats treated with femoral artery removal to induce hindlimb ischemia. The transplantation of 1×10^5^ SB623 cells into the ischemic adductor muscle seven days after ischemic induction resulted in significantly higher tissue perfusion in the SB623 group compared to control, with a significant increase in arteriogenesis and αSMA- and vWF-positive arterioles in the animals treated with SB623 cells. All animals were immunosuppressed with a daily administration of 10 mg/kg/day of cyclosporine [57].

MSCs have also been proposed as cellular therapeutics to deliver anti-tumour agents to tumour cells, such as IFN-beta (IFN-β). Non-clinical studies in syngeneic mouse tumours (ID8-R) and human xenograft (OVCAR3, SKOV3) ovarian tumour models transplanted intraperitoneally with murine MSCs stably expressing murine IFN-β showed that these cells could preferentially target the tumour sites and induce a significant reduction in tumour size compared to controls [58]. Although this study was carried out using MSCs transduced with a viral approach, the preliminary results on the efficacy of this product informed further studies supporting a FiH trial on patients with advanced ovarian cancer treated with MSCs genetically modified to express human IFN-β using a non-viral approach [50].

### 3.3. Toxicology

The toxicology assessment of genetically modified MSCs should identify, characterise, and quantify potential local and systemic toxicities following administration. The results from non-clinical proof-of-concept pharmacology studies should guide the design of the toxicology studies, considering animal species, ROA, therapeutic dose range, and dosing schedule. Since toxicity can be affected by the ROA, the site of administration, and the dosing schedule, studies should mimic the intended clinical scenario wherever possible. Additionally, if the genetically modified MSCs are intended for repeated dosing in the clinic, the same regimen should be used in the non-clinical studies.

Toxicology studies should assess acute and chronic toxicities, the reversibility of toxicities, delayed toxicities, and any dose-response effects. These studies should be designed to generate clinically meaningful and predictive data to support the safe use of the product in the intended clinical indication. Safety studies should be conducted in an appropriate pharmacologically relevant species and animals of both sexes should generally be investigated unless the clinical product is intended for a single sex and the regulatory authorities have agreed. The toxicology assessment, consisting of general toxicology endpoints (i.e., mortality, behavioural observations, body weights, and food/water consumption) should be provided to support the product safety profile. Relevant safety endpoints should include the histopathology of all major organs and tissues, serum chemistry, and haematology. Product specific pharmacodynamic biomarkers are also generally included for most ATMPs. These analyses may extend beyond single biomarkers where it is known that transgene products interact with the host endogenous cascade and have pleiotropic effects in whole animal systems. Focussed investigations of these effects are commonly included in toxicity studies; for example, immunotoxicology, neurotoxicity, or safety pharmacology endpoints to assess the risks to major physiological systems. These are usually only included where a clinical risk has been identified.

Disease-free animals are normally used for standard non-clinical pivotal toxicity studies to inform the safety of the candidate product in a study unconfounded by the presence of a disease. To minimise the risk of immune rejection of the transplanted human cells, immunocompromised or immunosuppressed animals can be used with appropriate controls. This approach should improve in vivo cell persistence, maximising the detection of any possible adverse effects. Toxicology studies should be performed under GLP [52,53,54], or otherwise justified and agreed with the regulatory authorities.

The toxicology programme for Alofisel included single-dose and repeat-dose toxicity studies performed in immunocompromised athymic nude rats. Several routes of administration were used, including the intended clinical route (perianal), as well as subcutaneous, intravenous, or intravaginal administrations. Single doses of 5 × 10^6^ or 10 × 10^6^ cells/rat were well tolerated in all ROAs used, except the intravenous administration group that was associated with the mortality of some of the animals dosed with the highest cell dose, related to pulmonary embolism [55].

In the case of genetically modified MSCs, it is important to consider the potential risks related to the over-expression of the transgene. To do so, the transgene(s) expressed by MSCs should be pharmacologically active in the animal species selected. If not, regulatory advice should be sought as to whether homologous species-specific transgenes should be used in the pivotal safety studies.

### 3.4. Biodistribution and Persistence

The investigation of biodistribution is particularly important for gene engineered MSC therapy products, as it helps to evaluate efficacy and anticipate potential safety issues related to unwanted distribution of the cells and/or transgene expression.

Generally, as already described for toxicology, the biodistribution assessment should be performed in animals of both sexes and should provide information on the distribution, persistence, and clearance of MSCs and possibly the expressed transgene at the site of administration and in all major organs, whether target or non-target tissues, preferably at different time points after administration. This approach allows any toxic effects observed (if any) to be correlated with the presence of the cell product and transgene expression. The administration route should mimic that intended for clinical use and the gene engineered MSC should be produced using a manufacturing process as close as possible to the final process intended for the clinic.

The characterisation of in vivo biodistribution in non-clinical studies can be performed in dedicated stand-alone studies or combined with toxicology studies. In both cases, the studies are usually performed to GLP unless otherwise justified [52,53,54]. In some instances, in vivo biodistribution can be included in the pharmacology studies in diseased animals to maximise the information obtained from those studies.

Biodistribution studies of candidate products should be detected using a sensitive assay, such as quantitative polymerase chain reaction (qPCR). For clinical trial approval and marketing authorisation, the methods of analysis used within the pivotal non-clinical safety study, including biodistribution, should be validated. Although the validation of the bioanalytical methods may not be strictly necessary before a FiH clinical study, sufficient information on the suitability of the used method, e.g., specificity and sensitivity (limit of detection) should be provided.

Safety and biodistribution studies should be designed on a case-by-case basis for each product, depending on the intrinsic features, such as the type of transgene(s) expressed, RoA, etc. However, the evaluation of how biodistribution studies in animals have been performed to characterise similar marketed products can help to identify the key studies that should be performed and how they can inform clinical translation.

For example, three biodistribution studies were performed for Alofisel in male and female athymic nude rats, using the intended clinical route and intravenous administration. The biodistribution of human eASCs was investigated by detecting human DNA in the major organs retrieved at different time points up to 6 months. When the clinical route was used, human DNA was detected up to 14 days after cell injection. When eASCs were administered intravenously, human genomic DNA was detected at a high level in the lungs of almost all treated athymic rats; however, this biodistribution profile is expected when using this ROA [55].

### 3.5. Tumourigenicity

Tumourigenicity is a major concern for all cell-based therapies, especially where stem and other multi-potent cells are used. Generally, unmodified MSCs are viewed as safe with cell abnormalities mainly arising during the cell manufacturing process. Genetic modifications can enhance the risk of tumourigenicity, particularly when integrating viral vectors are used. Whilst non-viral transfections do not necessarily integrate within the genome, the overexpression of a recombinant gene has the potential to lead to cellular transformations and thereby tumourigenicity. Results from the biodistribution/persistence studies on the duration of transgene over-expression should be considered when deciding whether long-term non-clinical studies to assess the tumourigenic potential of the candidate product are required.

Since the development of spontaneous cellular transformation can occur over a prolonged period, it is necessary to investigate tumourigenicity in vivo over a prolonged period, usually at least three to six months in an appropriate animal model, however, longer studies of up to twelve months are not uncommon.

Although Alofisel is not a genetically modified product, in vitro and in vivo studies to investigate tumourigenicity were included in the non-clinical strategy and represent a good example of some of the investigations that may be requested by the regulatory authorities. When cultured in vitro, eASCs reached a plateau in their growth curve by doubling to level 40, with most of the cell preparations becoming senescent between doubling levels 25 and 30, as confirmed by acidic β-galactosidase staining. No increases in telomerase activity or c-myc expression were observed during the process of expansion. In addition, eASC preparations showed no anchorage-independent growth in the soft-agar test and the karyotypes analysis did not show any signs of abnormality or transformation. In in vivo tumourigenicity tests, eASCs were injected subcutaneously into nude mice at various population doubling levels. All animals survived until the end of the study and no tumour-formation was observed. None of these studies indicated a risk of tumourigenicity, however a 5-year follow-up in patients has been requested by the regulators to further investigate the risks of ectopic tissue formation and tumourigenicity [55].

### 3.6. Immunogenicity

Although MSCs are not considered highly immunogenic, immunogenicity should be considered when designing non-clinical programmes. The consequences of immunogenicity in animal models are rarely translatable to clinical outcomes but understanding immunogenicity against MSCs and any expressed transgenes can assist in the interpretation of toxicology and biodistribution studies. For example, an immune response to the transgene product or MSCs themselves may be triggered in the non-clinical test species resulting in the reduced pharmacological activity of the product and hence a potential impact upon its safety and biodistribution. Whilst this does not translate to human risk, it may mean that the test model has not been suitably exposed to the ATMP being tested so possible safety or dose limiting effects could be missed.

In addition, where modifications have been made to the human wild-type transgene sequence there is a possibility that, in a clinical setting, these modifications may be seen as non-self by the host immune system. Again, it is not possible to translate this risk from non-clinical animal studies to human studies, but these data can be informative in the consideration of other more suitable investigations to mitigate this clinical risk. In vitro and in silico studies to predict the possible immunogenicity of neoantigens in modified transgenes can help to address some of these risks.

In vitro studies can also be helpful; however, they are not always fully predictive of the risks of immunogenicity in the clinic. In vitro studies were performed to investigate the immunogenicity of Alofisel, where eASCs were incubated with donor peripheral blood mononuclear cells (PBMCs), activated PBMCs, or conditioned supernatants from activated or non-activated PBMCs, and analysed for expression of MHC class I/II and co-stimulatory molecules on the cell surface. Although costimulatory molecules were not detected in any of these conditions, the MHC class I/II was upregulated when the eASCs were in contact with the activated PBMC or when they were in the presence of the conditioned supernatant of the activated PBMCs. Additionally, eASC expression of ligands binding NK cells was investigated in co-culture systems containing eASC and NK cells purified from donor PBMCs, and the NK cytotoxicity was assessed by NK cell degranulation and IFN-γ secretion. eASC expressed low levels of NK-cells activating ligands (CD94/NKG2A-B-C, HLA-I, MICA/B, ULBP-1/2/3, CD112, CD155, NKp30, and NKp46). Although IFN-γ release was observed in response to eASC, NK cell degranulation rates were very low. The clinical experience with Alofisel is still too limited to exclude risks of immunogenicity/allo-immunoreactions, therefore a post-authorisation safety study (PASS) follow-up is in place aimed at describing the long term safety observations including immunogenicity and tumourigenicity, with a final report expected by the EMA in 2029 [55].

## 4. Discussion

Non-clinical investigations of ATMPs are no longer a rarity; the last twenty years has seen an explosion in the number of cellular therapeutics in development and our understanding of both the regulatory expectations of risk mitigation and their clinical usage has increased dramatically. During that time, the technologies available to measure product specific biology have also developed exponentially. All of this means that informative studies can be designed that are able to generate clinically translatable data from non-clinical studies that increase the opportunity to improve clinical efficacy whilst reducing the safety risks associated with such complex biological products.

However, the successful translation of ATMPs from non-clinical to clinical studies still faces significant challenges These predominantly relate to the fast-evolving range of cell engineering and transfection technologies and the growing range of diseases being investigated for treatment with curative ATMPs.

In our efforts to design regulatory-compliant, ethical studies that assess the safety and efficacy of ATMPs it is critical that each product is considered individually. Wherever animal models are used to assess the safety and efficacy of ATMPs, the 3R’s (Reduction, Refinement and Replacement) must be considered during study design and execution. A single well-designed study including relevant clinically translatable endpoints is often more informative than multiple studies in non-relevant models. There are no prescriptive or generic approaches to non-clinical development of ATMPs, regular interaction with other researchers in the field and worldwide regulatory authorities are essential in developing safe and effective novel therapeutics.

## Figures and Tables

**Table 1:** Outline of the non-viral genetically modified MSC products investigated clinically.

Trial ID	Official Title	Trial Start	Trial End	Product Name	Phase	Status	Therapeutic Area	Indication	Delivery Method	Autologous/Allogeneic	Cell Source	Vector	Transfection Method	Gene Expressed
NCT01287936	A Phase I/IIa Study of the Safety and Efficacy of Modified Stromal Cells (SB623) in Patients with Stable Ischemic Stroke	2011	2015	SB623	I/II	Completed	Cardiovascular	Ischemic Stroke	Intracranial	Allogeneic	Bone marrow	Plasmid	Lipofectamine 2000	Intracellular domain of human Notch 1
NCT02416492	A Double-blind, Controlled Phase II Study of the Safety and Efficacy of Modified Stem Cells (SB623) in Patients with Chronic Motor Deficit from Traumatic Brain Injury (TBI)	2016	2019	SB623	II	Completed	Central Nervous System	Traumatic Brain Injury	Intracranial	Allogeneic	Bone marrow	Plasmid	Lipofectamine 2000	Intracellular domain of human Notch 1
NCT02448641	A Double-blind, Controlled Phase IIb Study of the Safety and Efficacy of Modified Stem Cells (SB-623) in Patients with Chronic Motor Deficit from Ischemic Stroke	2016	2018	SB623	II	Completed	Cardiovascular	Ischemic Stroke	Intracranial	Allogeneic	Bone marrow	Plasmid	Lipofectamine 2000	Intracellular domain of human Notch 1
NCT04524962	Phase I/IIA Study of Descartes-30 in Acute Respiratory Distress Syndrome	2020	-	Descartes-30	I/II	Ongoing, recruiting	Infectious Disease	Acute Respiratory Distress Syndrome/COVID-19	Undisclosed	Allogeneic	Undisclosed	mRNA	Undisclosed	Engineered to secrete two human DNases
NCT01977131	Phase I Study of Autologous Bone Marrow Stromal Cells with Modification by Hepatocyte Growth Factor to Treat Silicosis	2010	2013	MSC/HGF	I/II	Completed	Respiratory	Silicosis	Intravenous	Autologous	Bone marrow	Plasmid	Lipofectamine 2000	Hepatocyte growth factor
NCT02530047	PhaseI Study to Determine the Effects of Mesenchymal Stem Cells Secreting Interferon Beta in Patients with Advanced Ovarian Cancer	2016	2019	MSC-IFNβ	I	Completed	Oncology	Ovarian Cancer	Intraperitoneal	Allogeneic	Undisclosed	Plasmid	Electroporation	INFβ

## Data Availability

No data has been presented or analysed within this manuscript.
